# Grandparents - Grandchildren Relationship in Iran, 2017

**DOI:** 10.2174/1745017901814010296

**Published:** 2018-11-30

**Authors:** Yadollah Abolfathi Momtaz, Mahboube Mahdi Vidouje, Mahshid Foroughan, Robab Sahaf, Reza Laripour

**Affiliations:** 1 Iranian Research Center on Aging, University of Social Welfare and Rehabilitation Sciences, Tehran, Iran. Malaysian Research Institute on Ageing (MyAgeing), Universiti Putra Malaysia, Seri Kembangan, Selangor, Malaysia; 2 Iran University of Medical Sciences, Medical Education Research Center, Tehran, Iran

**Keywords:** Aged, Grandparent-grandchild relationship, Iran, Aging, Psychological, Sociodemographic

## Abstract

**Introduction:**

With the continuing growth of aged populations, it is imperative to find ways to maintain and improve the quality of life in old age. It has been documented that grandparents-grandchildren relationship is significantly contributed to quality of life of older adults. This study was conducted to identify the status and associated factors of grandparents-grandchildren relationship in a sample of Iran.

**Methods:**

This cross-sectional study was conducted on 377 community dwelling older adults 60 years and over living in Kashan, Iran. A multistage proportional random sampling technique was applied to obtain the sample. The grandparent -grandchildren relationship was measured by a researcher-developed 16-item scale. The data were analyzed using SPSS version 23 and AMOS 23.

**Results:**

The mean age of the respondents was 70.42(SD = 8.20) years. About 62% of the respondents were female and 60.7% were married. The average score of grandparents-grandchild relationship was 67.60(SD = 12.47). The multiple linear regression analysis revealed a significant model (F _(11, 365)_ = 19.05, *P* < 0.001), where information communication technology of grandparents, geographical distance between grandparents and grandchildren, and the quality of relationship between grandparents and parents of grandchildren were the most important predictors of the grandparents-grandchildren relationship.

**Conclusion:**

The findings from the current study showed that status of grandparents-grandchildren relationship is moderate to high and influenced by some factors. It is, therefore, suggested that policymakers pay more attention to strengthening grandparent-grandchild relationship by providing educational programs for families and encouraging the elderly to learn and use information communication technology.

## 1 INTRODUCTION

Aging is a continuous process, which can be studied from different aspects, including physical, psychological, and social dimensions [1]. The elderly population was not growing rapidly in the past, while today, due to health improvements and advances in knowledge and medical technologies, people have a longer life expectancy. Over the past five decades, rapid growth of the population, especially the elderly, and its consequences have been one of the most important problems in different societies, especially Asian countries [2, 3].

Previous reports have indicated that the world's aging population will increase from 200 million in 1950 to 1100 million in 2025 [1]. Currently, Iran, like other countries around the world, has undergone considerable changes in the age structure of its population as a result of reduced fertility and increased life expectancy in the past few decades [4, 5]. According to statistics, the proportion of the elderly population has increased from 6.6% in 1996 to 9.2% in 2016 [6]. 

Old age is a stage of life when an individual faces various problems, such as loneliness, diseases, and economic problems. These problems affect life satisfaction and quality of life of older adults [7]. The previous studies have found that grandparents-grandchildren relationship is one of the most important social determinants of health and life satisfaction at old age [8-10].

Several social and psychological theories of aging have been applied to conceptualize grandparents-grandchildren relationship. The solidarity theory is one of the most important theories, presented by Bengeston and colleagues. Six dimensions of “association”, “affection”, “consensus”, “resource sharing”, “strength of family norms”, and “opportunity structure for interaction”, are the essential aspects of intergenerational cohesion [11]. Another theory is the exchange theory in which social relations and reactions will be complete when involved people feel satisfied with the relationship [12]. As a result, elderly people, who may not have enough resources to contribute to intergenerational relationships, may feel disappointed. On the other hand, the modernization theory explains how the characteristics of modern societies have marginalized the elderly and diminished their influence and social involvement. In addition, old and young generations are being increasingly separated, especially in moral and intellectual aspects. It seems that with modernization of societies, the authority and importance of the elderly have decreased in the family and society. On the other hand, recent developments have led to changes in the family structure and family relations, such as grandparent-grandchild relationship, as confirmed in several studies [13, 14].

Iran, similar to many other countries around the world, is no exception to the process of modernization. It seems that the relationship between grandparents and grandchildren has deteriorated in Iranian families, which can lead to a generation gap in the Iranian society. According to the Madrid Operational Plan in 2002, the member states, including Iran, are responsible for identifying and strengthening intergenerational participation and relations with respect to intergenerational responsibilities [15].

Considering the importance of aging population, it is imperative to find ways to maintain and improve the quality of life of older adults [16]. In light of this consideration, several studies have found that quality of life of older adults is significantly influenced by grandparents-grandchildren relationship [9, 17]. Therefore, the current study was conducted to identify the status and associated factors of grandparents-grandchildren relationship in a sample of Iran.

## 2 MATERIALS AND METHODS

This cross-sectional study was conducted on a sample of older adults living in Kashan, Iran. Kashan is a historical city and part of Isfahan province. It is located in the center of Iran, 220 km south of Tehran. According to the census of 2016, the city population was 304 487, with the elderly comprising 10% of the population [18].

A multistage proportional random sampling technique was employed to obtain a sample of 377 community-dwelling older adults 60 years and older. The sample size with 95% confidence interval, 85% power, and attrition of 15%, was calculated 377 older persons.

At the first stage of the sampling procedure, the number of older adults covered by each health center was identified by health records. It is noteworthy to mention that community-dwelling older adults have health records at their respective health centers.

In the second stage, the sample size per health center was proportionally determined based on the total number of the elderly population covered by each health center and the required sample size.

At the last stage, health records were randomly selected from each center by the RAND function in Microsoft Excel. The selected numbers were being called until 385 responses were obtained. About 95% of the called numbers were agreed to participate. In case that selected number did not agree to participate in the study, another person would be randomly selected.

Data collection was conducted using a face-to-face interview by trained enumerators in respondents' homes, with a response rate of 98 percent. Fig. (**1**) shows the sampling process.

### 2.1 Ethics and Approvals

The study was approved by the University of Social Welfare and Rehabilitation Sciences and was in compliance with the Declaration of Helsinki and the World Medical Association guidelines. Oral informed consent was obtained from all respondents.

### 2.2 Measures

#### 2.2.1 Sociodemographic Factors

Sociodemographic characteristics of respondents including age, gender, level of education, economic status, employment status, marital status, housing status, individual health status, and geographical distance between grandparents and grandchildren were collected using self-report method.

#### 2.2.2 Grandparents-grandchildren Relationship

In this study grandparents-grandchildren relationship was measured using a researcher-developed 16-item scale. The scale was developed according to previous studies and Bengtson’s intergenerational solidarity model [10, 19]. The scale has three dimensions including associational solidarity (the frequency and patterns of interactional activities among family members), affection solidarity (the degree of positive sentiments between family members), and functional solidarity (the degree of support and exchange of resources among family members).

The associational solidarity dimension consisted of four questions. This dimension included questions such as, “How many times did your grandchildren come to visit you in the past year?” The affection dimension consisted of three questions such as, “How much do you love each other?” The functional dimension included nine questions. The extent of financial and social support of grandparents by grandchildren was measured by nine questions such as, “How many grandchildren help you move around the city (*e.g*., going to a doctor)?”

Face and content validity were established through an expert panel including gerontologists and psychologists. The items were scored on a 4-point Likert-scale. The scores were summed and transformed into a 0-100 scale with higher scores representing more relationship between grandparents and grandchildren. To make the total scores more informative, scores were categorized into tertiles as low (0 to 33.33), moderate (33.34 to 66.67) and high (66.68 to 100) levels.

#### 2.2.3 Statistical Analysis

To fulfill the study purposes, descriptive and inferential statistics using IBM SPSS version 23 and AMOS 23.0 (Analysis of Moment Structure) were performed. First, univariate and bivariate analyses were conducted to identify variables distribution and potential statistical associations between independent and dependent variables. Second, a multiple linear regression analysis was employed to find out the most important predictors of the grandparents-grandchildren relationship. The assumptions for multiple linear regression analysis such as homoscedasticity, linearity, and collinearity were examined and the results showed that the assumptions were satisfied.

The intercorrelations among study variables (Table **1**) and Variance Inflation Factors (VIF) were performed to determine multicollinearity. The results revealed no evidence of significant multicollinearity.

Results of psychometric properties of the scale

Construct validity was conducted by confirmed factor analysis using AMOS. The results from AMOS showed that model’s CMIN/df (2.51), GFI (.924), CFI (.921) and RMSEA (.063) fit statistics, indicated satisfactory model fit. Fig. (**2**) illustrates the model.

Internal consistency using Cronbach's alpha was used to determine the reliability of the scale and its subscales. The Cronbach's alpha for the total scale was found to be .86. Cronbach's alphas were acceptable for associational dimension (.65), affection dimension (.60), and functional dimension (.85).

## 3 RESULTS

The study sample consisted of 377 community-dwelling older adults 60 years and over, with the mean age of 70.42 ± 8.20 years. The majority of the respondents (68.4%) was 60-74 years old, and 71.6% were married. About half of the participants (42.2%) were no formal education. About two-thirds of the samples (60.5%), elderly pension and life partner were the most important sources of income. Moreover, 22.3% and 19.6% of the participants were pensioners and unemployed, respectively. Table **2** presents sociodemographic characteristics and health status of the study respondents. As can be seen from Table **2** more than two-thirds of the respondents (65.3%) reported their level of income as adequate, and about 93% owned a house.

The mean score of grandparents-grandchildren relationship was found to be 67.60 (SD = 12.47). A series of independent t-tests revealed no significant association between grandparent-grandchild relationship and gender (*P* = 0.813) and marital status (*P* = 0.068). Based on the findings, grandparents-grandchildren relationship scores were significantly different (*P* < 0.001) with respect to grandparents' information communication technology. The scores of subjects who did not have any skills were lower than those with full (*P* < 0.001) or moderate (*P* < 0.001) knowledge.

The Pearson’s correlation test showed a significant negative relationship between the grandparents' age and grandparent-grandchild relationship, whereas a significant positive relationship was found between grandparent-grandchild and grandparent-parent relationships. Two groups, including younger people (r = -0.24, *P* < 0.001) and those with a better relationship with parents (r = 0.48, *P* < 0.001), had a better relationship with their grandchildren (Table **1**).

However, Spearman’s correlation test showed a positive correlation between grandparent-grandchild relationship and grandparents’ income (*P* < 0.01; r, 0.15), while geographical distance showed a negative correlation with grandparent-grandchild relationship (*P* < 0.001; r = -0.19).

A multiple linear regression analysis was conducted to identify the overall grandparent-parent relationship predictors and the relative importance of each predictor. The results of multiple linear regression analysis showed that the following variables were major predictors of grandparent-grandchild relationship: grandparent-parent relationship, distance between grandparents and grandchildren, and grandparents' information communication technology. The analyses showed that these variables could predict 37% of variance in grandparent-grandchild relationship (F (11, 365) = 19.05, *P* < 0.001). The results are presented in Table **3**.

## 4 DISCUSSION

The purpose of the current study was to assess the status and related factors of grandparents-grandchildren relationship in a sample of Iranian older adults. The results showed grandparents-grandchildren relationship in Iran was found to be moderate to high. Around 45% of the respondents reported their relationship with their grandchildren as moderate. This finding is consistent with previous observations which found that the relationship between grandparents and grandchildren was moderate [10, 20, 21]. In contrast, some studies found Chinese grandparents have a close relationship with their grandchildren [22, 23].

Although some studies have shown that grandmothers have a better relationship with their grandchildren, as they are kind and form more intimate relationships with their family [10, 24-26], The findings from the present study revealed no significant association between the participants’ gender and grandparent-grandchild relationship.

Results of the present study showed a significant association between the level of education and grandparent-grandchild relationship and supported previous studies documented higher level of education of older adults improve grandparent-grandchild relationship [27, 28].

In the present study, the relationship between grandparents and grandchildren was not significantly different between married and unmarried grandparents. Contrary to the present findings, results from a study in China showed that married people tend to have more contact with their grandchildren [27].

The findings from the current study showed that the income level of grandparents is significantly contributed to more grandparent-grandchild relationship and supported previous studies found that grandparents with a higher income are more likely to have more effective relations and receive more support from their grandchildren [27, 29]. It seems that grandchildren are interested in having relationships with grandparents, who are financially privileged. Grandparents with a higher income and better economic status can attract children by giving those presents or other privileges.

Grandparents with good or excellent health reported better relations with their grandchildren since unhealthy people has limited abilities to spend time with their grandchildren. This finding is consistent with previous studies [10, 24].

Our study showed that the use of information and communication technology among grandparents is significantly contributed to a high relationship with their grandchildren. In line with our findings, some other studies have underlined the importance of technology to maintain the relationship between grandparents and grandchildren [30, 31].

The relation of grandparents with their grandchildren's parents was found to be a significant factor that is associated with grandparents-grandchildren relationship. This finding is consistent with previous studies that found the relation of grandparents with their adult children plays an important role in the grandparent-grandchild relationship [10, 24].

The finding from the current study showed that geographical distance is significant and inversely associated with grandparent-grandchild relationship and supported previous studies reported a greater geographical distance reduces the relationship between grandparents and grandchildren [27, 28, 32].

One of the limitations of this study is its cross-sectional design; therefore, longitudinal and interventional studies are suggested. In addition, this study only focused on the elderly population from Kashan. Therefore, the findings cannot be generalized to the entire Iranian population, and similar studies are recommended in other cities. Although psychometric properties of the grandparents-grandchildren relationship scale which developed by researchers were found to be valid, another limitation that should be addressed is related to the scale used to measure grandparents-grandchildren relationship which is a new scale.

## CONCLUSION

Despite the aforementioned limitations, to our knowledge, this is the first study to describe grandparents-grandchildren relationship in Iran. The results showed that the relationship between grandparents and grandchildren is moderate to high. The findings showed that information and communication technology skills, geographical distance, and the relation of grandparents with their grandchildren's parents (adult married children) are most important factors that are significantly associated with grandparents-grandchildren relationship. According to the findings from the present study, in order to enhance grandparents-grandchildren relationship, it is suggested that grandparents become acquainted with new technological and intellectual skills. In addition, families and adults children try to maintain their relationship with their parents.

## Figures and Tables

**Fig. (1):**
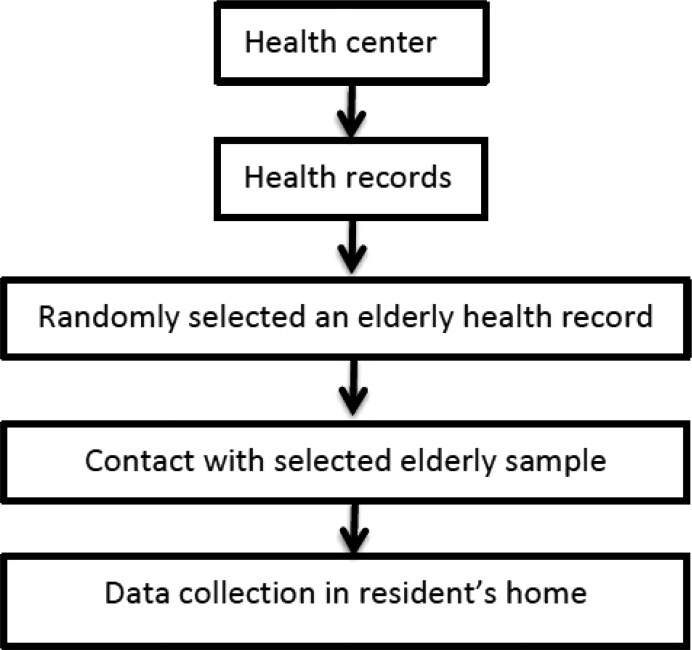
Flow chart of sampling process.

**Fig. (2):**
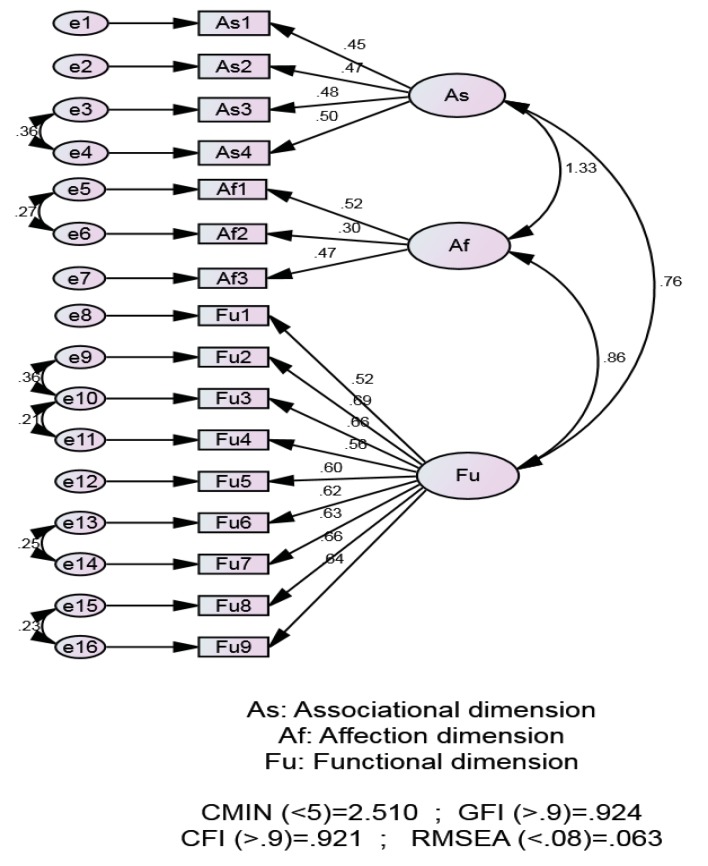
Confirmatory factor analysis model.

**Table 1:** The intercorrelations among study variables.

Variable	1	2	3	4	5	6	7	8	9	10	11	12
DV (1)		-.243**	.240**	0.10	.130*	-.152**	-.219**	-0.10	.497**	-.214**	.248**	.206**
Age (2)			-.315**	-.240**	-0.07	0.00	.483**	.505**	-.117*	0.10	-.239**	-.215**
Level of education (3)				.177**	.248**	-.188**	-.281**	-.227**	.160**	-0.02	.286**	.231**
Marital status (4)					.104*	-.110*	-.193**	-.202**	-0.09	0.01	.133**	0.06
Income (5)						-.312**	-.158**	-0.06	.180**	0.00	.194**	.207**
Subjective financial status (6)							.193**	-0.02	-.250**	-0.10	-0.05	-.182**
Employment status (7)								.346**	-.167**	-0.04	-.165**	-.378**
Number of grandchildren (8)									-0.03	0.07	-.226**	-.134**
Relation with parents (9)										-0.06	.110*	.168**
Distance (10)											.124*	0.04
ICT (11)												.281**
SRH (12)												
* p<.05, **p<.01 DV: dependent variable (grandparents-grandchildren relationship) ICT: information communication technology SRH: self-rated health												

**Table 2:** The profile of respondents by mean score of grandparent grandchildren relationship.

	Category	N	%	Mean	SD
Age	60-74	258	68.4	69.51	12.15
	75-84	93	24.7	63.34	12.29
	85+	26	6.9	63.81	12.03
Sex	Female	232	61.5	67.72	11.85
	Male	145	38.5	67.4	13.43
Employment status	Employed	41	10.9	65.91	14.04
	Retired	84	22.3	68.95	13.11
	Unemployed	252	66.8	67.42	11.98
Level of education	No Formal education	159	42.2	64.1	11.47
	Primary Education	149	39.5	68.11	11.47
	Secondary Education	69	18.3	74.55	13.73
Self-Rated Health	very good	9	2.4	65.86	18.47
	good	68	18	70.55	13.15
	moderate	190	50.4	69.2	11.02
	poor	110	29.2	63.14	12.8
Subjective financial status	Not enough	82	21.8	64.02	13.27
	Enough	246	65.3	68.43	11.98
	Saving	49	13	69.41	12.61
ICT	Never	339	89.9	66.56	11.74
	Somewhat	33	8.8	75.26	15.3
	Good	5	1.3	87.19	4.96
Total		377	100	67.60	12.47

**Table 3:** Results of multiple regression.

	B	SE	Beta	t
Constant	56.57	6.82		8.30
Age	-0.13	0.08	-0.09	-1.59
Education (Formal education)	1.67	1.19	0.07	1.40
Marital status (Married)	2.28	1.23	0.08	1.86
Employment status (Unemployed)	-1.75	1.64	-0.06	-1.07
Number of grandchildren	0.10	0.08	0.06	1.26
Relation with parents (Good)	7.06	0.72	0.44***	9.75
Distance	-1.82	0.37	-0.21***	-4.91
ICT (Using)	7.22	1.93	0.17***	3.75
Self-rated health (Good)	0.74	0.84	0.04	0.88
Subjective financial status (Not enough)	-0.74	1.41	-0.02	-0.53
Household income	-0.94	1.23	-0.03	-0.76
***p <.001 F(11, 365) = 19.05, P <. 001, R^2^ =.37				
